# Psychosocial experiences in IVF and spontaneous pregnancies: a comparative study

**DOI:** 10.1590/1980-220X-REEUSP-2026-0218en

**Published:** 2026-10-12

**Authors:** Kübra Nur Budak, Meltem Aydın Beşen

**Affiliations:** 1 Kahramanmaraş State Hospital, Kahramanmaraş, Türkiye.; 2 Mersin University, School of Health, Faculty of Health Sciences, Department of Midwifery, Mersin, Türkiye.

**Keywords:** Fertilization in Vitro, Psychological Well-Being, Stress, Psychological, Self-Compassion, Pregnancy, Fertilização in vitro, Bem-Estar Psicológico, Estresse Psicológico, Autocompaixão, Gravidez

## Abstract

**Objective:**

This study aimed to compare psychological well-being, perceived stress, and self-compassion in women who conceived via IVF and those with spontaneous pregnancies.

**Method:**

This descriptive, comparative cross-sectional study included 50 women who conceived via IVF and 100 who conceived spontaneously. Data were collected through face-to-face interviews between July 2024 and January 2025 using standardized scales. The Mann–Whitney U test, chi-square test, Kruskal–Wallis test, and Spearman correlation analysis were used.

**Results:**

Women in the IVF group had significantly higher perceived stress and lower psychological well-being and self-compassion than those with spontaneous pregnancies (p < 0.001). In the spontaneous pregnancy group, psychological well-being was positively associated with self-compassion and negatively associated with perceived stress.

**Conclusion:**

Women who conceived via IVF may benefit from comprehensive psychosocial support, particularly interventions aimed at enhancing self-compassion.

## RESUMO

**Objetivo:**

Comparar o bem-estar psicológico, o estresse percebido e a autocompaixão entre mulheres que conceberam por fertilização in vitro (FIV) e mulheres com gravidez espontânea.

**Método:**

Este estudo descritivo, comparativo e transversal incluiu 50 mulheres que conceberam por fertilização in vitro (FIV) e 100 mulheres com gravidez espontânea. Os dados foram coletados por meio de entrevistas presenciais entre julho de 2024 e janeiro de 2025, utilizando escalas padronizadas. Para a análise dos dados, foram utilizados o teste U de Mann–Whitney, o teste do qui-quadrado, o teste de Kruskal–Wallis e a análise de correlação de Spearman.

**Resultados:**

As mulheres do grupo FIV apresentaram níveis significativamente mais elevados de estresse percebido e níveis significativamente mais baixos de bem-estar psicológico e autocompaixão em comparação com aquelas do grupo de gravidez espontânea (*p* < 0,001). No grupo de gravidez espontânea, o bem-estar psicológico apresentou associação positiva com a autocompaixão e associação negativa com o estresse percebido.

**Conclusão:**

Mulheres que conceberam por fertilização in vitro podem beneficiar-se de um apoio psicossocial abrangente, especialmente de intervenções voltadas para o fortalecimento da autocompaixão.

## INTRODUCTION

Receiving an infertility diagnosis and undergoing treatment have not only biological but also psychosocial consequences for individuals and couples. The medical decision-making associated with infertility, uncertainty regarding treatment outcomes, repeated treatment failures, and the financial burden of costly procedures can lead to intense emotional turmoil^(1,2)^. During this period, couples may experience various psychological problems such as anxiety, depressive symptoms, stress, loneliness, and social isolation, all of which can adversely affect their psychological well-being^(1,3)^. It has been reported that the uncertainty experienced by women undergoing IVF treatment has a significant adverse effect on their psychological well-being. Uncertainty about whether conception will occur, heightened expectations regarding treatment outcomes, repeated attempts, and environmental factors may further exacerbate these effects^(4)^. Research also shows that high levels of stress and anxiety associated with infertility persist even after conception and that women who conceive following infertility treatment experience significantly greater anxiety than those who conceive spontaneously^(5,6,7)^. Factors such as the course of pregnancy, concerns about fetal health, and fear of miscarriage may further elevate stress levels in these women^(6)^.

Exposure to stress and emotional disturbances during pregnancy can adversely affect both maternal and fetal health^(8)^. In this context, self-compassion emerges as an important protective factor for psychological well-being. Self-compassion is defined as recognizing negative experiences such as pain, distress, inadequacy, or failure, accepting them without judgment, and acknowledging that such experiences are a natural part of human life^(9)^. The literature has shown that individuals with high levels of self-compassion demonstrate stronger stress-coping skills and greater psychological resilience^(10)^. Self-compassion is also recognized as a protective psychological resource for coping with psychosocial difficulties frequently encountered during infertility treatment, such as self-blame, feelings of inadequacy, and social pressure^(11)^. However, studies directly comparing levels of self-compassion and psychological well-being between women who conceive via IVF and those who conceive spontaneously remain scarce.

This study aimed to deepen understanding of the psychological dimensions of infertility and pregnancy and provide evidence-based insights for practitioners by comparing psychological well-being, perceived stress, and self-compassion between women who conceived via IVF and those who conceived spontaneously.

## METHOD

### Research Design

This descriptive, comparative cross-sectional study examined psychological well-being, perceived stress, and self-compassion in women who conceived via IVF and those who conceived spontaneously. Data were collected from each participant at a single time point.

### Study Population

The study population comprised women who conceived following a short IVF protocol and attended a private IVF center in Kahramanmaraş, Türkiye, as well as women who conceived spontaneously and attended the maternity outpatient clinic of a state hospital in Gaziantep, Türkiye. Data were collected between July 8, 2024, and January 31, 2025.

### Sample Size and Sampling

The sample size was calculated using data from a previous study^(7)^, with an effect size of 0.5033845, α = 0.05, and power = 0.80. Accordingly, the required sample size was determined to be 142 women, including 47 who conceived via IVF and 95 who conceived spontaneously. Ultimately, 150 women who met the eligibility criteria and agreed to participate were included in the study: 50 who conceived via IVF and 100 who conceived spontaneously. Participants were recruited using convenience sampling.

### Inclusion and Exclusion Criteria

The eligibility criteria for the IVF pregnancy group were as follows: conception following treatment with a short IVF protocol at a specialized IVF center; age 18 years or older; gestational age between 10 and 40 weeks; literacy in Turkish; and provision of voluntary consent to participate in the study.

Exclusion criteria included the presence of a diagnosed psychiatric disorder, communication barriers that could affect questionnaire completion, and conception through assisted reproductive technologies other than IVF.

The eligibility criteria for the spontaneous pregnancy group were as follows: follow-up at an antenatal clinic in a state hospital; age 18 years or older; spontaneous conception; gestational age between 10 and 40 weeks; literacy in Turkish; and voluntary agreement to participate in the study.

Exclusion criteria included a history of infertility treatment, conception through any assisted reproductive technology, and the presence of a diagnosed psychiatric disorder.

### Data Collection Instruments

Data were obtained using the Demographic Information Form, Psychological Well-Being Scale (PWBS), Perceived Stress Scale (PSS), and Self-Compassion Scale (SCS).

The Demographic Information Form included questions on participants’ sociodemographic characteristics, such as age, education, and employment status, and obstetric characteristics, such as gestational age, parity, and history of infertility.

PWBS: The scale was adapted into Turkish^(12)^. This 8-item scale yields scores ranging from 8 to 56, with higher scores indicating greater psychological well-being. The Cronbach’s alpha coefficient for the Turkish version is 0.80.

PSS: The scale was adapted into Turkish^(13)^. This 14-item, 5-point Likert-type scale measures perceived stress, with higher total scores indicating higher stress levels. The Cronbach’s alpha coefficient for the Turkish version is approximately 0.84.

SCS: This 24-item, 5-point Likert-type scale evaluates self-compassion, with higher scores reflecting higher self-compassion levels. The reported Cronbach’s alpha coefficient is 0.89^(14)^.

### Data Collection

Data were collected through face-to-face interviews conducted with participants in clinical rooms. After the researcher provided a detailed explanation of the study, the questionnaires were distributed, and participants were asked to complete them. The data collection process took approximately 10–15 minutes.

### Data Analysis

Data were analyzed using SPSS for Windows (version 25). The continuous variables were assessed for normality using the Kolmogorov–Smirnov test and were found to be non-normally distributed. Descriptive statistics, including means, standard deviations, minimum values, and maximum values, were used for continuous variables, while frequencies and percentages were used for categorical variables. The chi-square test was performed to compare categorical variables. The Mann–Whitney U test was used to compare two independent groups when the data were not normally distributed, and the Kruskal–Wallis test was used for comparisons involving three or more groups. Associations among scale scores and continuous variables were evaluated using Spearman’s rank correlation analysis. Statistical significance was set at p < 0.05.

### Ethical Considerations

The authors declare that the research presented in this manuscript was conducted in accordance with the ethical standards of the Clinical Research Ethics Committee of Mersin University (Approval No. 2024/299) and the 1964 Declaration of Helsinki and its subsequent amendments. All procedures involving human participants complied with these ethical principles. Written informed consent was obtained from all participants.

## RESULTS

Table 1 presents a comparison of the sociodemographic characteristics of women who conceived via IVF and those with spontaneous pregnancies. Significant differences were found between the groups with respect to age, partner age, and years of marriage; the IVF group was older and had been married longer (p < 0.05). With respect to education level, a higher proportion of participants in the spontaneous pregnancy group had completed high school or postgraduate education (p < 0.001). The employment rate was lower in the IVF group, and a statistically significant difference was observed in partners’ employment status (p < 0.05). Income level was significantly higher in the spontaneous pregnancy group (p = 0.029), and a higher proportion of participants in the IVF group resided in district centers (p = 0.024). No statistically significant differences were observed for the remaining variables.

**Table 1:** Sociodemographic characteristics of the study groups – Kahramanmaraş and Gaziantep, Türkiye, July 2024–January 2025.

Variables	IVF group (n = 50) Median (Min–Max)		Spontaneous pregnancy group (n = 100) Median (Min–Max)		Test statistic/p
Age	29 (22–39)		26 (19–40)		Z = −3.94; p < 0.001
Spouse’s Age	30 (25–42)		29 (22–44)		Z = −2.15; p = 0.032
Years of Marriage	4 (2–10)		3 (1–20)		Z = −3.76; p < 0.001
**Education**	**n**	**%** *	**n**	**%** *	
Literate	2	4.00	1	1.00	χ^2^ = 29.45; p < 0.001
Elementary School	24	48.00	16	16.00	
High School	23	46.00	48	48.00	
Graduate	1	2.00	35	35.00	
**Spouse’s Education**					
Literate	0	0.00	2	2.00	χ^2^ = 8.83; p = 0.032
Elementary School	2	4.00	3	3.00	
High School	33	66.00	42	42.00	
Graduate	15	30.00	53	53.00	
**Employment Status**					
Yes	5	10.00	40	40.00	χ^2^ = 14.29; p < 0.001
No	45	90.00	60	60.00	
**Spouse’s Employment Status**					
Yes	44	88.00	97	97.00	χ^2^ = 4.79; p = 0.029
No	6	12.00	3	3.00	
**Income Status**					
Budget Deficit	5	10.00	6	6.00	χ^2^ = 7.07; p = 0.029
Balanced Budget	27	54.00	35	35.00	
Budget Surplus	18	36.00	59	59.00	
**Social Security Status**					
Yes	50	100.00	93	93.00	χ^2^ = 3.67; p = 0.055
No	0	0.00	7	7.00	
**Residential Location**					
Provincial Center	19	38.00	22	22.00	χ^2^ = 7.44; p = 0.024
District Center	31	62.00	70	70.00	
Village	0	0.00	8	8.00	
**Family Structure**					
Nuclear Family	49	98.00	99	99.00	χ^2^ = 0.25; p = 0.615
Extended Family	1	2.00	1	1.00	
Total	50	100.00	100	100.00	

Z = standardized Mann–Whitney U test statistic; χ^2^ = chi-square test statistic;

*percentages were calculated by column.

Table 2 presents a comparison of perceived stress, psychological well-being, and self-compassion between women who conceived via IVF and those with spontaneous pregnancies. The median perceived stress score was significantly higher in the IVF group (47.5) than in the spontaneous pregnancy group (11) (U = −9.98, p < 0.001). The median psychological well-being score was significantly lower in the IVF group (41) than in the spontaneous pregnancy group (53) (U = −9.69, p < 0.001). Similarly, the median self-compassion score was significantly lower in the IVF group (62) than in the spontaneous pregnancy group (106) (U = −9.92, p < 0.001).

**Table 2:** Comparison of psychological outcomes between groups – Kahramanmaraş and Gaziantep, Türkiye, July 2024–January 2025.

Scales	IVF group (n = 50)	Spontaneous pregnancy group (n = 100)	Test statistic	p
	Median (Min–Max)	Median (Min–Max)	Z	p
PSS	47.5 (43–54)	11 (2–35)	−9.98	<0.001
PWBS	41 (26–52)	53 (43–56)	−9.69	<0.001
SCS	62 (51–70)	106 (66–114)	−9.92	<0.001

Z = standardized Mann–Whitney U test statistic.

A positive but nonsignificant association was observed between psychological well-being and perceived stress in women who conceived via IVF (r = 0.181, p = 0.208). Although a negative association was observed between psychological well-being and self-compassion, this association was not statistically significant (r = −0.270, p = 0.058). The association between perceived stress and self-compassion was negligible and nonsignificant (r = 0.05, p = 0.724) (Table 3).

**Table 3:** Correlations between PWBS, PSS, and SCS scores in the IVF group – Kahramanmaraş, Türkiye, July 2024–January 2025.

Scales (n = 50)	1		2		3	
	r	p	r	p	r	p
1. PWBS Total Score	–	–				
2. PSS Total Score	0.181	0.208	–	–		
3. SCS Total Score	−0.270	0.058	0.05	0.724	–	–

Spearman’s rank correlation.

A negative and statistically significant association was found between psychological well-being and perceived stress in women with spontaneous pregnancies (r = −0.35, p < 0.001), indicating that higher perceived stress is associated with lower psychological well-being. A positive and statistically significant association was observed between psychological well-being and self-compassion (r = 0.30, p = 0.003), indicating that higher levels of psychological well-being were associated with higher levels of self-compassion. A strong negative association was found between perceived stress and self-compassion (r = −0.54, p < 0.001), suggesting that higher self-compassion is significantly associated with lower perceived stress (Table 4).

**Table 4:** Correlations between PWBS, PSS, and SCS scores in the spontaneous pregnancy group – Gaziantep, Türkiye, July 2024– January 2025.

Scales (n = 100)	1		2		3	
	r	p	r	p	r	p
1. PWBS Total Score	–	–				
2. PSS Total Score	−0.35	<0.001	–	–		
3. SCS Total Score	0.30	0.003	−0.54	<0.001	–	–

Spearman’s rank correlation.

## DISCUSSION

The present study aimed to examine associations among psychological well-being, perceived stress, and self-compassion in women who conceived via IVF and those who conceived spontaneously. Comparison of the sociodemographic characteristics of women who conceived via IVF and those with spontaneous pregnancies revealed statistically significant differences in several variables. The older age of women in the IVF group may be attributable to the tendency to seek IVF treatment later in life. The literature also reports that the incidence of infertility increases with age and that fertility decreases in women over the age of 30. This finding underscores the importance of monitoring the risks associated with advanced maternal age in IVF pregnancies and providing adequate psychological support and counseling services. The findings also showed that women in the IVF group had lower levels of education. Previous studies have reported that educational level is associated with increased health literacy and fertility awareness, as well as a tendency to delay pregnancy until later in life. The relationship between educational level and fertility and health-seeking behaviors may therefore be bidirectional^(15,16)^.

The IVF process has been reported to impose substantial physical and emotional burdens on women, leading to difficulties in their working lives and a tendency to leave the workforce^(17)^. In this study, the employment rate was lower in the IVF group; therefore, it is important for midwifery services to prioritize guidance on work–life balance and the development of stress-management skills. Furthermore, the findings indicated that the majority of women in the IVF group lived in peripheral regions. The geographic distribution of health services is a key determinant of individuals’ health-related decision-making processes^(18)^. Increasing access to midwifery services in underserved or peripheral regions and developing remote counseling programs to reduce geographic inequalities may improve access to professional support for women who conceive via IVF.

The study found that women who conceived via IVF had significantly higher perceived stress scores than those who conceived spontaneously. This may be attributable to the multifaceted stressors inherent in the IVF process, including uncertainty, repeated medical interventions, substantial financial burdens, social expectations, and low levels of self-compassion^(5,19)^. These findings are consistent with previous studies demonstrating similar patterns. Previous research has found that women who became pregnant following IVF had higher anxiety levels than those who conceived spontaneously and suggested that this difference may be related to traumatic experiences associated with the treatment process^(7)^. Similarly, a large-scale cohort study indicated that women who conceived through ART reported higher levels of anxiety and stress during early pregnancy than women with spontaneous pregnancies^(20)^.

These findings suggest that the psychological effects of the IVF process may persist throughout pregnancy. The current study further revealed that women who conceived via IVF had significantly lower levels of psychological well-being than those who conceived spontaneously. It was reported that women who conceived through IVF carried feelings of loss, grief, and powerlessness into their pregnancy; feelings of helplessness and uncertainty were particularly prominent, and these women reported a strong need for reassurance within the healthcare system^(21)^. These findings show that the psychological effects of IVF treatment continue during pregnancy and place women in a more emotionally vulnerable position. Similarly, a longitudinal study indicated that psychological health was poorest during the first trimester and the second postpartum month; pregnancy-related stress and social support were among its strongest predictors^(22)^. These results suggest that women who conceive via IVF may have limited resources for coping with stress and may benefit from personalized psychological support programs. The literature also emphasizes that women with IVF pregnancies may struggle to find meaning, report lower life satisfaction, be adversely affected by their living conditions, and exhibit depressive symptoms^(23)^. When these findings are considered collectively, the evidence suggests that women with IVF pregnancies require monitoring not only biologically but also psychologically and socially.

The findings showed that women who conceived through IVF had lower levels of self-compassion than those who conceived spontaneously. The literature reports that self-compassion is a protective factor in coping with emotional stress related to infertility and that individuals with high levels of self-compassion exhibit greater psychological resilience^(9)^. Previous research has reported low levels of self-compassion and high levels of anxiety and depression among infertile women. The same study also reported a negative association between self-compassion and depression and anxiety^(24)^. Similarly, another study reported that self-compassion mediates the relationship between marital quality and psychological distress in infertile women^(25)^. These results indicate that self-compassion is a protective factor that supports psychological resilience and coping with stress throughout the infertility process. Therefore, healthcare professionals should implement self-compassion-based interventions in the care of women who conceive via IVF.

The present study found that women who conceived through IVF reported higher levels of perceived stress and lower levels of self-compassion and psychological well-being than women who conceived spontaneously. However, no significant associations were identified among these variables within the IVF group. The IVF process is often accompanied by multiple psychological and social challenges, including the financial burden of treatment, concerns regarding pregnancy loss, anxiety about fetal health, and the social expectations associated with fertility and motherhood^(26)^. These factors may create a complex emotional context in which the relationships between stress, self-compassion, and psychological well-being are less straightforward than expected. Although not statistically significant, the positive association observed between psychological well-being and perceived stress in the IVF group is noteworthy. Women who achieve pregnancy following infertility treatment may simultaneously experience feelings of relief, hope, and personal fulfillment while continuing to face concerns about the continuation and outcome of the pregnancy. Therefore, psychological well-being and perceived stress may coexist during this period despite the inverse association commonly reported in the literature. In addition, self-compassion is influenced by various individual and contextual factors, including cultural values, self-­perception, previous life experiences, and social support systems^(27)^. The combined influence of these factors may have contributed to the absence of significant relationships among the study variables. Furthermore, the relatively small size of the IVF group may have limited the ability to detect statistically significant associations. Future studies with larger samples and longitudinal designs are needed to further explore the complex interactions among stress, self-compassion, and psychological well-being in women who conceive through IVF.

Mindfulness-based programs, emotional support interventions, and self-compassion-based approaches are recommended to enhance psychological well-being, reduce stress, and improve self-compassion^(10)^. When implemented collaboratively by midwives and psychologists, these approaches may enhance women’s psychological resilience by reducing pregnancy-related anxiety^(28,29)^. Holistic support addressing both physical and psychological health among women with IVF pregnancies may improve maternal and infant outcomes. The literature emphasizes that self-compassion helps maintain psychological balance by facilitating emotional regulation in infertile women^(30)^. In the current study, the significant associations among psychological well-being, perceived stress, and self-compassion in women with spontaneous pregnancies indicate that self-compassion serves as a protective factor in emotional regulation during spontaneous pregnancy.

## CONCLUSION

The present study examined associations among psychological well-being, perceived stress, and self-compassion in women with spontaneous pregnancies and those who conceived through IVF. The findings indicate that women who conceived through IVF exhibited higher levels of perceived stress and, conversely, lower levels of self-compassion and psychological well-being. In women with spontaneous pregnancies, psychological well-being was found to enhance stress-coping abilities, with self-compassion playing a protective role in this process. These findings suggest that psychological processes vary depending on pregnancy type and that supporting self-compassion is particularly important in IVF pregnancies.

Women with IVF pregnancies require specialized monitoring that addresses biological, psychological, and social factors. Strengthening self-compassion is critical for managing stress effectively, maintaining psychological well-being, and supporting mother–infant bonding. In this regard, it may be beneficial to implement self-compassion-based approaches, emotional support strategies, and awareness programs within midwifery practices. Comprehensive care models involving collaboration between midwives and psychologists may strengthen the psychological health of women who conceive through IVF and contribute to more positive childbirth experiences.

### Strengths and Limitations

A key strength of this study is the direct comparison of psychological well-being, perceived stress, and self-compassion between women who conceived through IVF and those with spontaneous pregnancies using standardized and comparable measurement instruments. Additional strengths include the use of power analysis to determine the sample size and the recruitment of more participants than the initially estimated sample size. However, several limitations should be acknowledged. The cross-sectional design precludes causal inference; moreover, the use of convenience sampling limits the generalizability of the findings. The single-center design further limits the generalizability of the findings to diverse socioeconomic and cultural populations.

## Data Availability

The entire dataset supporting the results of this study was published in the article itself.
