# Supplementary material for: Psychosocial experiences in IVF and spontaneous pregnancies: a comparative study
Source: Rev Esc Enferm USP. 2026 Oct 12;60:e20260218. doi: 10.1590/1980-220X-REEUSP-2026-0218en (PMC13671634; doi:10.1590/1980-220X-REEUSP-2026-0218en)
Supplement: Translation

# Translation: Experiencias psicosociales en embarazos por fecundación in vitro y embarazos espontáneos: un estudio comparativo

**DOI:** 10.1590/1980-220X-REEUSP-2026-0218es

**Authors:** Kübra Nur Budak, Meltem Aydın Beşen

**Affiliations:** 1 Kahramanmaraş State Hospital, Kahramanmaraş, Türkiye.; 2 Mersin University, School of Health, Faculty of Health Sciences, Department of Midwifery, Mersin, Türkiye.

**Keywords:** Fecundación in vitro, Bienestar Psicológico, Estrés Psicológico, Autocompasión, Embarazo

## Abstract

**Objetivo:**

Comparar el bienestar psicológico, el estrés percibido y la autocompasión entre mujeres que concibieron mediante fecundación in vitro (FIV) y mujeres con embarazo espontáneo.

**Método:**

Este estudio descriptivo, comparativo y transversal incluyó a 50 mujeres que concibieron mediante fecundación in vitro (FIV) y a 100 mujeres con embarazo espontáneo. Los datos se recopilaron mediante entrevistas presenciales entre julio de 2024 y enero de 2025, utilizando escalas estandarizadas. Para el análisis de los datos se emplearon la prueba U de Mann–Whitney, la prueba de chi-cuadrado, la prueba de Kruskal–Wallis y el análisis de correlación de Spearman.

**Resultados:**

Las mujeres del grupo de FIV presentaron niveles significativamente más altos de estrés percibido y niveles significativamente más bajos de bienestar psicológico y autocompasión en comparación con las mujeres del grupo de embarazo espontáneo (*p* < 0,001). En el grupo de embarazo espontáneo, el bienestar psicológico mostró una asociación positiva con la autocompasión y una asociación negativa con el estrés percibido.

**Conclusión:**

Las mujeres que concibieron mediante fecundación in vitro pueden beneficiarse de un apoyo psicosocial integral, especialmente de intervenciones dirigidas a fortalecer la autocompasión.

## INTRODUCCIÓN

Recibir un diagnóstico de infertilidad y someterse a tratamiento tiene consecuencias no solo biológicas, sino también psicosociales para las personas y las parejas. La toma de decisiones médicas relacionadas con la infertilidad, la incertidumbre respecto a los resultados del tratamiento, los repetidos fracasos terapéuticos y la carga financiera de los costosos procedimientos pueden provocar una intensa agitación emocional^(1,2)^. Durante este período, las parejas pueden experimentar diversos problemas psicológicos, como ansiedad, síntomas depresivos, estrés, soledad y aislamiento social, los cuales pueden afectar negativamente su bienestar psicológico^(1,3)^. Se ha informado que la incertidumbre que experimentan las mujeres que se someten a un tratamiento de fecundación in vitro (FIV) tiene un efecto adverso significativo en su bienestar psicológico. La incertidumbre sobre si se producirá la concepción, las expectativas elevadas respecto a los resultados del tratamiento, los intentos repetidos y los factores ambientales pueden exacerbar aún más estos efectos^(4)^. Las investigaciones también muestran que los altos niveles de estrés y ansiedad asociados con la infertilidad persisten incluso después de la concepción, y que las mujeres que conciben tras un tratamiento de infertilidad experimentan una ansiedad significativamente mayor que aquellas que conciben de manera espontánea^(5,6,7)^. Factores como el curso del embarazo, las preocupaciones sobre la salud fetal y el miedo a un aborto espontáneo pueden elevar aún más los niveles de estrés en estas mujeres^(6)^.

La exposición al estrés y a las alteraciones emocionales durante el embarazo puede afectar negativamente tanto la salud materna como la fetal^(8)^. En este contexto, la autocompasión se perfila como un importante factor protector para el bienestar psicológico. La autocompasión se define como el reconocimiento de experiencias negativas, tales como el dolor, la angustia, la sensación de insuficiencia o el fracaso; su aceptación sin juzgarlas; y el reconocimiento de que dichas experiencias son una parte natural de la vida humana^(9)^. La literatura ha demostrado que las personas con altos niveles de autocompasión muestran mayores habilidades para afrontar el estrés y una mayor resiliencia psicológica^(10)^. La autocompasión también se reconoce como un recurso psicológico protector para lidiar con las dificultades psicosociales que suelen surgir durante el tratamiento de la infertilidad, como la autoculpa, los sentimientos de insuficiencia y la presión social^(11)^. Sin embargo, siguen siendo escasos los estudios que comparan directamente los niveles de autocompasión y bienestar psicológico entre las mujeres que conciben mediante FIV y aquellas que conciben de manera espontánea.

El objetivo de este estudio fue profundizar en la comprensión de las dimensiones psicológicas de la infertilidad y el embarazo, y proporcionar información basada en la evidencia a los profesionales de la salud mediante la comparación del bienestar psicológico, el estrés percibido y la autocompasión entre las mujeres que concibieron mediante FIV y aquellas que concibieron de manera espontánea.

## MÉTODO

### Diseño de la Investigación

Este estudio transversal, descriptivo y comparativo examinó el bienestar psicológico, el estrés percibido y la autocompasión en mujeres que concibieron mediante FIV y aquellas que lo hicieron de manera espontánea. Los datos se recolectaron de cada participante en un único momento.

### Población del Estudio

La población del estudio estuvo compuesta por mujeres que concibieron tras un protocolo corto de FIV y acudieron a un centro privado de FIV en Kahramanmaraş, Turquía, así como por mujeres que concibieron de manera espontánea y acudieron a la clínica ambulatoria de maternidad de un hospital público en Gaziantep, Turquía. Los datos se recolectaron entre el 8 de julio de 2024 y el 31 de enero de 2025.

### Tamaño de la Muestra y Muestreo

El tamaño de la muestra se calculó utilizando datos de un estudio previo^(7)^, con un tamaño del efecto de 0,5033845, α = 0,05 y poder = 0,80. En consecuencia, se determinó que el tamaño de muestra requerido era de 142 mujeres, incluidas 47 que concibieron mediante FIV y 95 que concibieron de manera espontánea. Finalmente, se incluyeron en el estudio 150 mujeres que cumplían con los criterios de elegibilidad y aceptaron participar: 50 que concibieron mediante FIV y 100 que concibieron de manera espontánea. Las participantes fueron reclutadas mediante un muestreo por conveniencia.

### Criterios de Inclusión y Exclusión

Los criterios de elegibilidad para el grupo de embarazos por FIV fueron los siguientes: concepción tras un tratamiento con un protocolo corto de FIV en un centro especializado en FIV; tener 18 años o más; edad gestacional entre 10 y 40 semanas; saber leer y escribir en turco; y dar su consentimiento voluntario para participar en el estudio.

Los criterios de exclusión incluyeron la presencia de un trastorno psiquiátrico diagnosticado, barreras de comunicación que pudieran afectar la respuesta al cuestionario y la concepción mediante tecnologías de reproducción asistida distintas de la FIV.

Los criterios de elegibilidad para el grupo de embarazos espontáneos fueron los siguientes: seguimiento en una clínica prenatal de un hospital público; edad de 18 años o más; concepción espontánea; edad gestacional entre 10 y 40 semanas; saber leer y escribir en turco; y consentimiento voluntario para participar en el estudio.

Los criterios de exclusión incluyeron antecedentes de tratamiento de infertilidad, concepción mediante cualquier tecnología de reproducción asistida y la presencia de un trastorno psiquiátrico diagnosticado.

### Instrumentos de Recolección de Datos

Los datos se obtuvieron mediante el Formulario de Información Demográfica, la Escala de Bienestar Psicológico (Psychological Well-Being Scale - PWBS), la Escala de Estrés Percibido (Perceived Stress Scale - PSS) y la Escala de Autocompasión (Self-Compassion Scale - SCS).

El Formulario de Información Demográfica incluía preguntas sobre las características sociodemográficas de las participantes, tales como edad, nivel educativo y situación laboral, así como características obstétricas, como la edad gestacional, la paridad y los antecedentes de infertilidad.

PWBS: La escala se adaptó al turco^(12)^. Esta escala de 8 ítems arroja puntuaciones que van de 8 a 56, donde las puntuaciones más altas indican un mayor bienestar psicológico. El coeficiente alfa de Cronbach para la versión turca es de 0,80.

PSS: La escala se adaptó al turco^(13)^. Esta escala de 14 ítems, tipo Likert de 5 puntos, mide el estrés percibido; las puntuaciones totales más altas indican niveles de estrés más elevados. El coeficiente alfa de Cronbach para la versión turca es de aproximadamente 0,84.

SCS: Esta escala de 24 ítems, tipo Likert de 5 puntos, evalúa la autocompasión; las puntuaciones más altas reflejan niveles más altos de autocompasión. El coeficiente alfa de Cronbach reportado es de 0,89^(14)^.

### Recolección de Datos

Los datos se recopilaron mediante entrevistas presenciales realizadas con los participantes en consultorios clínicos. Después de que el investigador proporcionara una explicación detallada del estudio, se distribuyeron los cuestionarios y se pidió a los participantes que los completaran. El proceso de recolección de datos duró aproximadamente entre 10 y 15 minutos.

### Análisis de Datos

Los datos se analizaron utilizando SPSS para Windows (versión 25). Se evaluó la normalidad de las variables continuas mediante la prueba de Kolmogorov-Smirnov y se determinó que no seguían una distribución normal. Para las variables continuas se utilizaron estadísticas descriptivas, incluyendo medias, desviaciones estándar, valores mínimos y valores máximos, mientras que para las variables categóricas se utilizaron frecuencias y porcentajes. Se realizó la prueba de chi-cuadrado para comparar las variables categóricas. Se utilizó la prueba U de Mann–Whitney para comparar dos grupos independientes cuando los datos no seguían una distribución normal, y la prueba de Kruskal–Wallis para comparaciones que involucraban tres o más grupos. Las asociaciones entre las puntuaciones de las escalas y las variables continuas se evaluaron mediante el análisis de correlación de rangos de Spearman. La significación estadística se fijó en p < 0,05.

### Consideraciones Éticas

Los autores declaran que la investigación presentada en este manuscrito se llevó a cabo de conformidad con las normas éticas del Comité de Ética en Investigación Clínica de la Universidad de Mersin (N.º de aprobación 2024/299) y la Declaración de Helsinki de 1964 y sus enmiendas posteriores. Todos los procedimientos que involucraron a participantes humanos cumplieron con estos principios éticos. Se obtuvo el consentimiento informado por escrito de todas las participantes.

## RESULTADOS

La Tabla 1 presenta una comparación de las características sociodemográficas de las mujeres que concibieron mediante FIV y aquellas con embarazos espontáneos. Se encontraron diferencias significativas entre los grupos con respecto a la edad, la edad de la pareja y los años de matrimonio; el grupo de FIV era de mayor edad y llevaba más tiempo casado (p < 0,05). En cuanto al nivel educativo, una mayor proporción de participantes del grupo de embarazos espontáneos había completado la preparatoria o estudios de posgrado (p < 0,001). La tasa de empleo fue menor en el grupo de FIV, y se observó una diferencia estadísticamente significativa en la situación laboral de las parejas (p < 0,05). El nivel de ingresos fue significativamente mayor en el grupo de embarazos espontáneos (p = 0,029), y una mayor proporción de participantes del grupo de FIV residía en centros de distrito (p = 0,024). No se observaron diferencias estadísticamente significativas para las variables restantes.

**Tabla 1:** Características sociodemográficas de los grupos de estudio – Kahramanmaraş y Gaziantep, Turquía, Julio de 2024–Enero de 2025.

Variables	Grupo de FIV (n = 50) Mediana (mín.–máx.)		Grupo de embarazos espontáneos (n = 100) Mediana (mín.–máx.)		Prueba estadística/p
Edad	29 (22–39)		26 (19–40)		Z = −3,94; p < 0,001
Edad del cónyuge	30 (25–42)		29 (22–44)		Z = −2,15; p = 0,032
Años de matrimonio	4 (2–10)		3 (1–20)		Z = −3,76; p < 0,001
**Nivel educativo**	**n**	**%** *	**n**	**%** *	
Alfabetizados	2	4,00	1	1,00	χ^2^ = 29,45; p < 0,001
Escuela primaria	24	48,00	16	16,00	
Bachillerato	23	46,00	48	48,00	
Posgrado	1	2,00	35	35,00	
**Estudios del cónyuge**					
Sabe leer y escribir	0	0,00	2	2,00	χ^2^ = 8,83; p = 0,032
Escuela primaria	2	4,00	3	3,00	
Instituto	33	66,00	42	42,00	
Posgrado	15	30,00	53	53,00	
**Situación laboral**					
Sí	5	10,00	40	40,00	χ^2^ = 14,29; p < 0,001
No	45	90,00	60	60,00	
**Situación laboral del cónyuge**					
Sí	44	88,00	97	97,00	χ^2^ = 4,79; p = 0,029
No	6	12,00	3	3,00	
**Situación de ingresos**					
Déficit presupuestario	5	10,00	6	6,00	χ^2^ = 7,07; p = 0,029
Presupuesto equilibrado	27	54,00	35	35,00	
Superávit presupuestario	18	36,00	59	59,00	
**Situación en materia de Seguridad Social**					
Sí	50	100,00	93	93,00	χ^2^ = 3,67; p = 0,055
No	0	0,00	7	7,00	
**Ubicación residencial**					
Centro provincial	19	38,00	22	22,00	χ^2^ = 7,44; p = 0,024
Centro de distrito	31	62,00	70	70,00	
Pueblo	0	0,00	8	8,00	
**Estructura familiar**					
Familia nuclear	49	98,00	99	99,00	χ^2^ = 0,25; p = 0,615
Familia extensa	1	2,00	1	1,00	
Total	50	100,00	100	100,00	

Z = estadística estandarizada de la prueba U de Mann-Whitney; χ^2^ = estadística de la prueba de chi-cuadrado;

*los porcentajes se calcularon por columnas.

La Tabla 2 presenta una comparación del estrés percibido, el bienestar psicológico y la autocompasión entre las mujeres que concibieron mediante FIV y aquellas con embarazos espontáneos. La mediana de la puntuación de estrés percibido fue significativamente mayor en el grupo de FIV (47,5) que en el grupo de embarazos espontáneos (11) (U = −9,98, p < 0,001). La mediana de la puntuación de bienestar psicológico fue significativamente menor en el grupo de FIV (41) que en el de embarazos espontáneos (53) (U = −9,69; p < 0,001). Del mismo modo, la mediana de la puntuación de autocompasión fue significativamente más baja en el grupo de FIV (62) que en el grupo de embarazos espontáneos (106) (U = −9,92; p < 0,001).

**Tabla 2:** Comparación de los resultados psicológicos entre los grupos – Kahramanmaraş y Gaziantep, Turquía, Julio de 2024–Enero de 2025.

Escalas	Grupo de FIV (n = 50)	Grupo de embarazos espontáneos (n = 100)	Prueba estadística	p
	Mediana (mín.-máx.)	Mediana (mín.–máx.)	Z	p
PSS	47,5 (43–54)	11 (2–35)	−9,98	<0,001
PWBS	41 (26–52)	53 (43–56)	−9,69	<0,001
SCS	62 (51–70)	106 (66–114)	−9,92	<0,001

Z = estadística estandarizada de la prueba U de Mann-Whitney.

Se observó una asociación positiva, aunque no significativa, entre el bienestar psicológico y el estrés percibido en las mujeres que concibieron mediante FIV (r = 0,181; p = 0,208). Aunque se observó una asociación negativa entre el bienestar psicológico y la autocompasión, esta asociación no fue estadísticamente significativa (r = −0,270; p = 0,058). La asociación entre el estrés percibido y la autocompasión fue insignificante y no significativa (r = 0,05, p = 0,724) (Tabla 3).

**Tabla 3:** Correlaciones entre las puntuaciones de PWBS, PSS y SCS en el grupo de FIV – Kahramanmaraş, Turquía, Julio de 2024–Enero de 2025.

Escalas (n = 50)	1		2		3	
	r	p	r	p	r	p
1. Puntuación total de la PWBS	–	–				
2. Puntuación total del PSS	0,181	0,208	–	–		
3. Puntuación total del SCS	−0,270	0,058	0,05	0,724	–	–

Correlación de rangos de Spearman.

### Correlación de Rangos de Spearman

Se observó una asociación negativa y estadísticamente significativa entre el bienestar psicológico y el estrés percibido en mujeres con embarazos espontáneos (r = −0,35, p < 0,001), lo que indica que un mayor estrés percibido se asocia con un menor bienestar psicológico. Se observó una asociación positiva y estadísticamente significativa entre el bienestar psicológico y la autocompasión (r = 0,30; p = 0,003), lo que indica que unos niveles más altos de bienestar psicológico se asociaban con unos niveles más altos de autocompasión. Se encontró una fuerte asociación negativa entre el estrés percibido y la autocompasión (r = −0,54, p < 0,001), lo que sugiere que una mayor autocompasión se asocia significativamente con un menor estrés percibido (Tabla 4).

**Tabla 4:** Correlaciones entre las puntuaciones de PWBS, PSS y SCS en el grupo de embarazos espontáneos – Gaziantep, Turquía, Julio de 2024–Enero de 2025.

Escalas (n = 100)	1		2		3	
	r	p	r	p	r	p
1. Puntuación total de la PWBS	–	–				
2. Puntuación total del PSS	−0,35	<0,001	–	–		
3. Puntuación total del SCS	0,30	0,003	−0,54	<0,001	–	–

Correlación de rangos de Spearman.

## DISCUSIÓN

El presente estudio tuvo como objetivo examinar las asociaciones entre el bienestar psicológico, el estrés percibido y la autocompasión en mujeres que concibieron mediante FIV y aquellas que concibieron de manera espontánea. La comparación de las características sociodemográficas de las mujeres que concibieron mediante FIV y las que tuvieron embarazos espontáneos reveló diferencias estadísticamente significativas en varias variables. La mayor edad de las mujeres del grupo de FIV podría atribuirse a la tendencia a buscar tratamiento de FIV en una etapa más avanzada de la vida. La literatura también indica que la incidencia de la infertilidad aumenta con la edad y que la fertilidad disminuye en las mujeres mayores de 30 años. Este hallazgo subraya la importancia de monitorear los riesgos asociados con la edad materna avanzada en los embarazos por FIV y de brindar apoyo psicológico y servicios de asesoramiento adecuados. Los resultados también mostraron que las mujeres del grupo de FIV tenían niveles educativos más bajos. Estudios previos han señalado que el nivel educativo está asociado con una mayor alfabetización en salud y conciencia sobre la fertilidad, así como con una tendencia a posponer el embarazo hasta una etapa más tardía de la vida. Por lo tanto, la relación entre el nivel educativo y la fertilidad, así como los comportamientos de búsqueda de atención médica, podría ser bidireccional^(15,16)^.

Se ha informado que el proceso de FIV impone cargas físicas y emocionales sustanciales a las mujeres, lo que genera dificultades en su vida laboral y una tendencia a abandonar la fuerza laboral^(17)^. En este estudio, la tasa de empleo fue menor en el grupo de FIV; por lo tanto, es importante que los servicios de partería den prioridad a la orientación sobre el equilibrio entre el trabajo y la vida personal y al desarrollo de habilidades para el manejo del estrés. Además, los resultados indicaron que la mayoría de las mujeres del grupo de FIV vivían en regiones periféricas. La distribución geográfica de los servicios de salud es un determinante clave de los procesos de toma de decisiones relacionadas con la salud de las personas^(18)^. Aumentar el acceso a los servicios de partería en regiones desatendidas o periféricas y desarrollar programas de asesoramiento a distancia para reducir las desigualdades geográficas podría mejorar el acceso al apoyo profesional para las mujeres que conciben mediante FIV.

El estudio reveló que las mujeres que quedaron embarazadas mediante FIV presentaban puntuaciones de estrés percibido significativamente más altas que aquellas que quedaron embarazadas de manera espontánea. Esto puede atribuirse a los factores estresantes multifacéticos inherentes al proceso de FIV, entre los que se incluyen la incertidumbre, las intervenciones médicas repetidas, las cargas financieras sustanciales, las expectativas sociales y los bajos niveles de autocompasión^(5,19)^. Estos hallazgos concuerdan con estudios previos que demostraron patrones similares. Investigaciones anteriores han encontrado que las mujeres que quedaron embarazadas tras una FIV presentaban niveles de ansiedad más altos que aquellas que concibieron de manera espontánea, y sugirieron que esta diferencia podría estar relacionada con experiencias traumáticas asociadas al proceso de tratamiento^(7)^. De manera similar, un estudio de cohorte a gran escala indicó que las mujeres que concibieron mediante técnicas de reproducción asistida (TRA) reportaron niveles más altos de ansiedad y estrés durante las primeras etapas del embarazo que las mujeres con embarazos espontáneos^(20)^.

Estos hallazgos sugieren que los efectos psicológicos del proceso de FIV pueden persistir a lo largo del embarazo. El presente estudio reveló además que las mujeres que concibieron mediante FIV presentaban niveles significativamente más bajos de bienestar psicológico que aquellas que concibieron de manera espontánea. Se informó que las mujeres que concibieron mediante FIV arrastraban sentimientos de pérdida, duelo e impotencia hacia su embarazo; los sentimientos de desamparo e incertidumbre eran particularmente prominentes, y estas mujeres manifestaron una fuerte necesidad de recibir apoyo y tranquilidad por parte del sistema de salud^(21)^. Estos hallazgos muestran que los efectos psicológicos del tratamiento de FIV continúan durante el embarazo y colocan a las mujeres en una posición emocionalmente más vulnerable. De manera similar, un estudio longitudinal indicó que la salud psicológica era más deficiente durante el primer trimestre y el segundo mes posparto; el estrés relacionado con el embarazo y el apoyo social se encontraban entre sus predictores más fuertes^(22)^. Estos resultados sugieren que las mujeres que conciben mediante FIV pueden tener recursos limitados para lidiar con el estrés y podrían beneficiarse de programas de apoyo psicológico personalizados. La literatura también destaca que las mujeres con embarazos por FIV pueden tener dificultades para encontrarle sentido a su vida, reportan una menor satisfacción con la vida, se ven afectadas negativamente por sus condiciones de vida y presentan síntomas depresivos^(23)^. Al considerar estos hallazgos en conjunto, la evidencia sugiere que las mujeres con embarazos por FIV requieren ser monitoreadas no solo biológicamente, sino también psicológicamente y socialmente.

Los hallazgos mostraron que las mujeres que concibieron mediante FIV tenían niveles más bajos de autocompasión que aquellas que concibieron de manera espontánea. La literatura indica que la autocompasión es un factor protector para afrontar el estrés emocional relacionado con la infertilidad y que las personas con altos niveles de autocompasión muestran una mayor resiliencia psicológica^(9)^. Investigaciones previas han reportado bajos niveles de autocompasión y altos niveles de ansiedad y depresión entre las mujeres infértiles. El mismo estudio también reportó una asociación negativa entre la autocompasión y la depresión y la ansiedad^(24)^. De manera similar, otro estudio indicó que la autocompasión media la relación entre la calidad de la relación conyugal y el malestar psicológico en mujeres con infertilidad^(25)^. Estos resultados indican que la autocompasión es un factor protector que favorece la resiliencia psicológica y la capacidad de afrontar el estrés a lo largo del proceso de infertilidad. Por lo tanto, los profesionales de la salud deberían implementar intervenciones basadas en la autocompasión en la atención a las mujeres que conciben mediante FIV.

El presente estudio encontró que las mujeres que concibieron mediante FIV reportaron niveles más altos de estrés percibido y niveles más bajos de autocompasión y bienestar psicológico que las mujeres que concibieron de manera espontánea. Sin embargo, no se identificaron asociaciones significativas entre estas variables dentro del grupo de FIV. El proceso de FIV suele ir acompañado de múltiples desafíos psicológicos y sociales, entre ellos la carga financiera del tratamiento, las preocupaciones relacionadas con la pérdida del embarazo, la ansiedad por la salud del feto y las expectativas sociales asociadas con la fertilidad y la maternidad^(26)^. Estos factores pueden crear un contexto emocional complejo en el que las relaciones entre el estrés, la autocompasión y el bienestar psicológico son menos directas de lo esperado. Aunque no es estadísticamente significativa, la asociación positiva observada entre el bienestar psicológico y el estrés percibido en el grupo de FIV es digna de mención. Las mujeres que logran un embarazo tras un tratamiento de infertilidad pueden experimentar simultáneamente sentimientos de alivio, esperanza y realización personal, al tiempo que siguen enfrentándose a preocupaciones sobre la continuidad y el resultado del embarazo. Por lo tanto, el bienestar psicológico y el estrés percibido pueden coexistir durante este período a pesar de la asociación inversa que se suele reportar en la literatura. Además, la autocompasión se ve influida por diversos factores individuales y contextuales, entre ellos los valores culturales, la autopercepción, las experiencias de vida previas y los sistemas de apoyo social^(27)^. La influencia combinada de estos factores pudo haber contribuido a la ausencia de relaciones significativas entre las variables del estudio. Además, el tamaño relativamente pequeño del grupo de FIV pudo haber limitado la capacidad de detectar asociaciones estadísticamente significativas. Se necesitan estudios futuros con muestras más grandes y diseños longitudinales para explorar más a fondo las complejas interacciones entre el estrés, la autocompasión y el bienestar psicológico en las mujeres que conciben mediante FIV.

Se recomiendan programas basados en la atención plena, intervenciones de apoyo emocional y enfoques basados en la autocompasión para mejorar el bienestar psicológico, reducir el estrés y aumentar la autocompasión^(10)^. Cuando se implementan de manera colaborativa por parte de parteras y psicólogos, estos enfoques pueden mejorar la resiliencia psicológica de las mujeres al reducir la ansiedad relacionada con el embarazo^(28,29)^. Un apoyo integral que aborde tanto la salud física como la psicológica en las mujeres con embarazos por FIV podría mejorar los resultados maternos e infantiles. La literatura destaca que la autocompasión ayuda a mantener el equilibrio psicológico al facilitar la regulación emocional en mujeres con infertilidad^(30)^. En el presente estudio, las asociaciones significativas entre el bienestar psicológico, el estrés percibido y la autocompasión en mujeres con embarazos espontáneos indican que la autocompasión actúa como un factor protector en la regulación emocional durante el embarazo espontáneo.

## CONCLUSIÓN

El presente estudio examinó las asociaciones entre el bienestar psicológico, el estrés percibido y la autocompasión en mujeres con embarazos espontáneos y en aquellas que concibieron mediante FIV. Los resultados indican que las mujeres que concibieron mediante FIV mostraron niveles más altos de estrés percibido y, por el contrario, niveles más bajos de autocompasión y bienestar psicológico. En las mujeres con embarazos espontáneos, se observó que el bienestar psicológico mejoraba la capacidad para afrontar el estrés, y que la autocompasión desempeñaba un papel protector en este proceso. Estos hallazgos sugieren que los procesos psicológicos varían según el tipo de embarazo y que fomentar la autocompasión es particularmente importante en los embarazos por FIV.

Las mujeres con embarazos por FIV requieren un monitoreo especializado que aborde los factores biológicos, psicológicos y sociales. Fortalecer la autocompasión es fundamental para manejar el estrés de manera efectiva, mantener el bienestar psicológico y apoyar el vínculo madre-bebé. En este sentido, podría ser beneficioso implementar enfoques basados en la autocompasión, estrategias de apoyo emocional y programas de concientización en las prácticas de partería. Los modelos de atención integral que impliquen la colaboración entre parteras y psicólogos podrían fortalecer la salud psicológica de las mujeres que conciben mediante FIV y contribuir a experiencias de parto más positivas.

### Fortalezas y Limitaciones

Una fortaleza clave de este estudio es la comparación directa del bienestar psicológico, el estrés percibido y la autocompasión entre las mujeres que concibieron mediante FIV y aquellas con embarazos espontáneos, utilizando instrumentos de medición estandarizados y comparables. Otras fortalezas incluyen el uso de un análisis de poder para determinar el tamaño de la muestra y el reclutamiento de más participantes que el tamaño de muestra estimado inicialmente. Sin embargo, deben reconocerse varias limitaciones. El diseño transversal impide la inferencia causal; además, el uso de un muestreo por conveniencia limita la generalizabilidad de los hallazgos. El diseño unicéntrico limita aún más la generalizabilidad de los hallazgos a poblaciones con diversidad socioeconómica y cultural.

## Data Availability

El conjunto completo de datos que respalda los resultados de este estudio se publicó en el propio artículo.
